# Glycemic trajectories of fasting blood glucose on the pathological responses in breast cancer women with and without concomitant diabetes

**DOI:** 10.1371/journal.pone.0319314

**Published:** 2026-03-05

**Authors:** Saba Javed, Umar Javed, Dzul Azri Mohamed Noor, Nur Hafzan Md Hanafiah, Tanveer Mustafa, Anees ur Rehman, Sabariah Noor Harun

**Affiliations:** 1 School of Pharmaceutical Sciences, Universiti Sains Malaysia, USM, Penang, Malaysia; 2 Department of Pharmacy Practice, Faculty of Pharmaceutical Sciences, Government College University, Faisalabad, Pakistan; 3 Gujranwala Institute of Nuclear Medicine & Radiotherapy (GINUM), Gujranwala, Pakistan; 4 Department of Pharmacy Practice, Faculty of Pharmacy, Bahauddin Zakariya University Multan, Multan, Pakistan; Faculty of Pharmaceutical sciences Times University Multan, PAKISTAN

## Abstract

**Background:**

Most existing studies have predominantly examined the impact of single blood glucose measurements without considering how fluctuations over time may influence clinical outcomes. Moreover, no studies have yet explored the effect of blood glucose trajectories on pathological responses in breast cancer patients undergoing neo-adjuvant chemotherapy (NACT), highlighting a significant gap in the current literature.

**Purpose:**

To determine the impact of dynamic changes in blood glucose during neo-adjuvant chemotherapy on the pathological responses in women with breast cancer, with and without concomitant diabetes.

**Method:**

This prospective cohort study was conducted among women with locally advanced breast cancer receiving treatment at GINUM Hospital in Gujranwala, Pakistan. Data for the prospective phase were collected from 20 January 2023 to–13 May 2024. Clinical data were obtained using a structured data collection form. Diabetes status was confirmed at baseline using Glycated hemoglobin (HbA1c) and Fasting blood glucose (FBG) tests. Patients were classified into glycemic trajectories based on the values of their FBG values during the treatment period.

**Results:**

Five blood glucose trajectories were identified from the start of breast cancer treatment to the post-treatment period: normal glycemic trajectory (23 patients, 4.1%), erratic glycemic trajectory (130 patients, 23.2%), consistently hyperglycemic trajectory (127 patients, 22.7%), controlled diabetes (130 patients, 23.2%), and uncontrolled diabetes (150 patients, 26.8%). All glycemic trajectory groups demonstrated a non-significant increase in the odds of achieving Pathological partial response (pPR). Similar to pPR, the various categories of blood glucose trajectories were associated with a non-significant increase in the odds of Pathological no response (pNR). Among nondiabetic patients the group I(normal) and group III (consistently hyperglycemic) trajectories showed no significant interaction with pathological responses, as indicated by repeated measures ANOVA.

**Conclusion:**

Our findings indicated that fluctuations in FBG levels among individuals with diabetes were not associated with pathological responses to neo-adjuvant chemotherapy. This suggests that the rise in blood glucose levels in diabetic patients are more likely driven by their pre-existing metabolic dysfunction rather than their chemotherapy related pathological responses.

## 1. Introduction

Cancer refers to a collection of diseases marked by the unchecked growth and spread of abnormal cells, which may form tumors or infiltrate surrounding tissues and organs, leading to damage and dysfunction. These rogue cells can develop in nearly any area of the body and may be triggered by various genetic, environmental, and lifestyle factors. Some of the most prevalent types of cancer are breast cancer, lung cancer, prostate cancer, colorectal cancer, and skin cancer [1]. In 2020, breast cancer was the most commonly diagnosed cancer in women, accounting for 11.7% of all cancer cases [2]. Incidence of breast cancer are rising in Asia contributing to a global burden of disease. One in nine women in Pakistan have been found to have breast cancer [3]. Diabetes and breast cancer are two significant chronic diseases, with diabetes affecting approximately 16% of breast cancer patients [4]. The presence of diabetes in breast cancer patients can influence treatment regimens and alter responses to chemotherapy [5].

Fasting blood glucose (FBG) can reflect short-term changes in blood glucose levels, which is particularly useful during chemotherapy when patients may experience rapid fluctuations in blood glucose due to the effects of drugs, changes in diet, stress, and other factors [6]. FBG measurements can capture these short-term variations more effectively than HbA1c, which reflects an average of over 2–3 months. Moreover, FBG provides immediate feedback on the current glucose status, which is crucial for making timely adjustments to treatment regimens, dietary interventions, and other management strategies during chemotherapy. Chemotherapy can induce acute changes in blood glucose levels, including hyperglycemia or hypoglycemia, which are important to detect and manage promptly [7]. Repeated FBG measurements can help identify these acute changes and allow rapid intervention. Unlike HbA1c, which depends on the lifespan of red blood cells. FBG measurements are not influenced by factors such as anemia, transfusions, or marrow suppression, which can affect red blood cell turnover and potentially lead to misleading HbA1c results [8].

Investigating the impact of blood glucose trajectories or variabilities on treatment outcomes provides crucial insights, as group-based trajectories capture dynamic changes over time, offering clinicians invaluable information about the progression of chronic diseases and treatment responses [9]. However, studies focusing on these trajectories in breast cancer are notably lacking. Most studies have predominantly examined the impact of single blood glucose measurements without considering how fluctuations over time might influence outcomes. Additionally, no studies have yet explored the effect of blood glucose trajectories on pathological response in breast cancer patients following neo-adjuvant chemotherapy (NACT), highlighting a significant gap in the current literature.

The objective of this study was to determine the impact of dynamic changes in blood glucose during neo-adjuvant chemotherapy on the pathological responses in breast cancer women with and without concomitant diabetes. A prospective cohort study is necessary to comprehend the impact of dynamic changes in blood glucose on the pathological responses in women with and without concomitant diabetes who have been diagnosed with breast cancer. Assessing the impact of blood glucose trajectories on the pathological response is expected to provide a more precise representation of current glucose levels.

## 2. Material and methods

### 2.1. Study design

A prospective cohort study was conducted on women with locally advanced breast cancer undergoing treatment at GINUM Hospital in Gujranwala, Pakistan.

### 2.2. Study population

Patients meeting the inclusion criteria were consecutively enrolled in the study from 20 January 2023 to 13 May 2024, with data documented using a predesigned data collection form.

#### 2.2.1. Inclusion criteria.

- locally advanced breast cancer women with and without concomitant diabetes.
- Age 18–65 years.
- Patient at the start of the chemotherapy cycle. All patients received the same dose of chemotherapy: taxane plus anthracycline, and for HER2 breast cancer women (trastuzumab along with taxane)

#### 2.2.2. Exclusion criteria.

- Patients with stage 1 or stage 4 cancer, those lost to follow-up.
- women with breast cancer who had mental disorders, were pregnant or lactating, renal dialysis patients, and patients using anti-psychotic medication that affects glucose levels.

Informed consent was obtained from each participant before their inclusion in the study, ensuring ethical compliance throughout the study.

### 2.3. Ethical statement

This study obtained ethical approval from the ethical review board of Gujranwala Institute of Nuclear Medicine and Radiotherapy (GINUM), Pakistan, with the letter reference No.GNM-AD-RCD-06–85 (ERB) and the Jawatankuasa Etika Penyelidikan Manusia Universiti Sains Malaysia (JEPeM-USM) approval with the study protocol code USM/JEPeM/PP/22080542. All participants provided written informed consent.

### 2.4. Data collection and examined variables

Diabetes status was also confirmed by attending physicians while taking the patient’s medical history. Further, the diabetes status was confirmed by the HbA1c and FBG tests performed at baseline.

The HbA1c test was conducted before the chemotherapy cycle began, after the chemotherapy cycle ended, and after the last follow-up. The FBG test was performed after an overnight fast of 8 or more hours. The FBG test was conducted regularly before each cycle of chemotherapy over a period of six months, comprising a total of eight cycles.

The diagnosis of diabetes status was confirmed by taking FBG and HbA1c values at baseline and following the standard definition recommended by the World Health Organization (WHO) for diabetes patients. The baseline diagnosis of diabetes is defined as fasting plasma glucose >7.0mmol/L (126 mg/dL) and 2-h post-load plasma glucose >11.1mmol/L (200 mg/dL), or HbA1c>6.5% (48 mmol/L), or random blood glucose >11.1mmol/L (200 mg/dL) [10]. Inform consent was obtained from each of the participants.

Patients were classified into five groups of glycemic trajectories based on the values of their FBG tests. Trajectories were categorized based on descriptions from previous studies [7,11,12]

- **Group I (**Normal glycemic trajectory) Among the breast cancer women without diabetes were classified as having normal glycemic if seven or more FBG test results were normal (<109mg/dL) during the chemotherapy cycle.
- **Group II** (Erratic glycemic instability) Among the breast cancer women without diabetes, they were classified as having erratic glycemic instability if two or more alterations in the glycemic status are > 126mg/dL (i.e., having impaired glucose tolerance, fluctuating among having diabetes from the start of treatment to the end of the chemotherapy cycle.
- **Group III (**Consistently hyperglycemia) Among the breast cancer women without diabetes, they were classified as having consistently hyperglycemia if seven or more FBG test results were >109mg/dL during the chemotherapy cycle.
- **Group IV** (Uncontrolled diabetes) Glycemic control among patients with diabetes was defined as poor or uncontrolled if the participant’s FBG test results were above or equal to 180 mg/dL [13].
- **Group V (**Controlled diabetes) is defined as the HbA1c value under 7% and FBG test results were less than or equal to 160 mg/dL [12].

### 2.5. Measurement of outcomes

The primary measured outcomes in this study were the postoperative pathological responses. After 3–4 weeks of NACT, most patients underwent modified radical mastectomy surgery. Pathological responses were then checked after two months of follow-up. Tumor tissue was surgically removed after NACT, and a pathological examination was conducted.

This assessment provides valuable information about the extent of tumor cell death (necrosis) and the degree of tumor regression. Pathologists evaluate factors like the percentage of viable tumor cells, fibrosis, and inflammation, which classify the tumor’s response to chemotherapy. The main goal of NACT efficacy evaluation is to achieve pathological complete response (pCR) [14]. The responses were defined according to the latest 7^th^ edition of the American Joint Committee on Cancer (AJCC) criteria and categorized into pathological complete response (pCR), pathological partial response (pPR), and pathological no response (pNR) [15]. pCR was defined as the complete removal of breast carcinoma cells and lymph nodes by pathologically resected tissue after NACT [16].pPR was defined as the decrease in either or both tumor size and lymph node with the pretreatment tumor size and lymph node. pNR was defined as no changes in either tumor size and lymph node with the pretreatment tumor size and lymph node [17].

### 2.6. Breast cancer stages

Breast cancer stages can be defined as the combination of tumor (T), nodes (N), and distant metastases (M) category designations. It is the most clinically useful staging system developed by the AJCC; based on T, N, and M classification, the stages can be categorized into 4 stages as described in Table 1. Stage 0 is defined as carcinoma in situ (Tis) [18].

**Table 1:** Breast cancer staging.

Stages	T stage	N stage	M stage
**Stage 0**	Tis	N0	M0
**Stage1**			
IA	T1	N0	M0
IB	T0-T1	N1	M0
**Stage 2**			
IIA	T0-T1	N0-N1	M0
IIB	T2-T3	NO-N1	M0
**Stage 3**			
IIIA	T0-T3	N2	M0
IIIB	T4	N0-N2	M0
IIIC	Any T	N3	M0

Abbreviation: Tis = Carcinoma in situ, T0 = No evidence of primary tumor, T1-T4 = a higher category means increasing in tumor size, N0 = No regional lymph node, N1-N3 = increasing the regional nodal group, M0 = no evidence of metastasis

## 3. Data analysis

Categorical variables were presented as frequencies and percentages. Continuous variables were presented as mean ±SD if normally distributed, and as median with interquartile range if non-normally distributed. The Kolmogorov-Smirnov test was used to assess the normality of the data distribution. The Chi-square test and Fisher’s exact test were applied to examine the association between diabetes status and variables such as age groups, body mass index, body surface area, socioeconomic status, marital status, educational level, employment status, comorbidities, clinical characteristics, and pathological responses.

A general linear model (GLM) repeated measure ANOVA was used to evaluate i) interaction effect between blood glucose value and pathological responses and (ii) dynamic changes of FBG over time. In this study, we created a series of different dummy variables specifying the trajectory of each patient included in the study. These variables were included in the multi-nominal regression models (Erratic trajectory as the reference) to assess associations of trajectories with the pathological responses. For the categorical comparisons, age ≤ 50 years, normal weight, Clinical stage IIA, and the Luminal A molecular subtype were used as reference group. All statistical analysis was performed using SPSS software (version 23), while data visualization was conducted using R statistical software.

## 4. Results

### 4.1. Demographic characteristics

Age, BMI, and blood glucose trajectories were significantly associated with all pathological response categories (p = 0.029, p = 0.001, p = 0.008), as shown in Table 2. However, no significant association was found between Body surface area (BSA), marital status, educational level, employment status, and diabetes status with pathological responses (all p > 0.05) (Table 2).

**Table 2:** Comparison of demographic characteristics between pathological responses (N = 560).

Variables at diagnosis	Total (N = 560)	pCR N = 142)	pPR (N = 290)	pNR (N = 128)	p-values
**Age(years) Mean±SD**	46 ± 9.1	45 ± 9.5	47 ± 9.2	48 ± 9.2	**0.009**
**Age group**					**0.029**
≤50 years	351(62.7)	102(71.8)	175(60.3)	74(57.8)	
>50 years	209(37.3)	40(28.2)	115(39.7)	54(42.2)	
**BMI**					**<0.001**
Normal weight	129(23.0)	37(26.1)	76(26.2)	16(12.5)	
Overweight	175(31.1)	47(33.1)	94(32.4)	34(26.6)	
Obese	256(45.7)	58(40.8)	120(41.1)	78(60.9)	
**BSA**					0.098
<1.64	231(41.3)	67(47.2)	120(41.1)	44(34.4)	
1.64-1.75	129(23.0)	36(25.4)	60(20.7)	33(25.8)	
1.75-2.0	200(35.7)	39(27.5)	110(37.9)	51(39.8)	
**Socio-economic status**					0.312
Lower class	236(42.1)	60(42.3)	115(39.7)	61(47.7)	
Lower middle class	324(57.9)	82(57.7)	175(60.3)	67(52.3)	
**Marital status**					0.349
Divorced	12(2.1)	3(2.1)	5(1.7)	4(3.1)	
Married	499(89.1)	123(86.6)	266(91.7)	110(85.9)	
Unmarried	41(7.3)	15(10.6)	15(5.2)	11(8.6)	
Widow	8(1.4)	1(0.7)	4(1.4)	3(2.3)	
**Educational level**					0.140
Illiterate	64(11.4)	16(11.3)	36(12.4)	12(9.4)	
Primary	90(16.1)	21(14.8)	51(17.6)	18(14.1)	
Secondary	297(53.0)	70(49.3)	161(55.5)	66(51.6)	
Graduate	109(19.5)	35(24.6)	42(14.5)	32(25.0)	
**Employment status**					0.77
Unemployed	549(98)	140(98.6)	281(96.9)	128(100.0)	
Employed	11(2.0)	2(1.4)	9(3.1)	0(0.0)	
**Diabetes status**					0.189
Diabetes	280(50.0)	67(47.2)	140(48.3)	73(57.0)	
Non-diabetes	280(50.0)	75(52.8)	150(51.7)	55(43.0)	
**Trajectories Groups**					**0.008**
Group I	23(4.1)	1(0.7)	15(5.2)	7(5.5)	
Group II	130(23.2)	42(29.6)	67(23.1)	21(16.4)	
Group III	127(22.7)	32(22.5)	68(23.4)	27(21.1)	
Group V	130(23.2)	27(19.0)	59(20.3)	44(34.4)	
Group IV	150(26.8)	40(28.2)	81(27.9)	29(22.7)	

Abbreviations: BMI = Body mass index, BSA = Body surface area, pCR = pathological complete response, pPR = pathological partial response, pNR = pathological no response, SD = standard deviation, Lower class = 12000–20,000 PKR, Lower middle class = 20,000–60,000.

### 4.2. Predictors of pathological responses in breast cancer women with and without concomitant diabetes

We developed a multi-nominal regression model to determine the predictors of pathological responses among breast cancer women with and without concomitant diabetes. Pathological responses were used as the dependent variables in the model, and demographic and clinical characteristics were used as independent variables.

**Fig 1:**
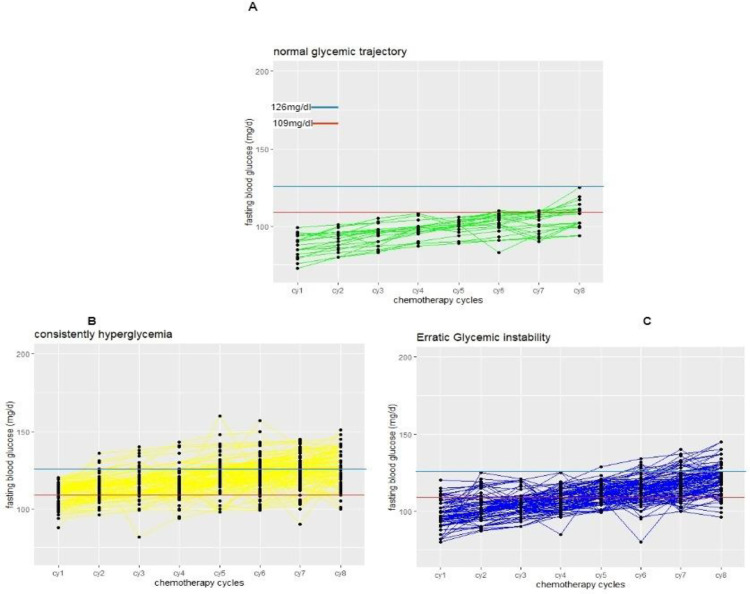
Glycemic trajectories in breast cancer women without diabetes after receiving neo-adjuvant chemotherapy.

Using pathological complete response (pCR) as the reference for the dependent variable in Table 3, clinical stage IIIB was found to significantly increase the odds of achieving a partial pathological response (pPR) after neo-adjuvant chemotherapy (NACT), with an odds ratio (OR) of 2.71 (95% CI: 1.22–5.99). Additionally, patients with Human epidermal growth factor receptor 2 (HER2-positive) and triple-negative breast cancer (TNBC) molecular subtypes showed a significant reduction in the odds of achieving pPR. Interestingly, all categories of blood glucose trajectories were associated with a non-significant increase in the odds of attaining pPR. In contrast, patients aged over 50 years had a higher likelihood of achieving a pathological non-response (pNR) after NACT, with an OR of 1.90 (95% CI: 1.11–3.24). Obesity significantly increased the odds of pNR, with an OR of 2.81 (95% CI: 1.36–5.80). Moreover, clinical stage IIIC was strongly associated with an increased likelihood of pNR, with an OR of 5.83 (95% CI: 1.85–18.30). Similar to pPR, the various categories of blood glucose trajectories were associated with no significant increase in the odds of pNR, as detailed in Table 3 and Fig 1.

**Table 3:** Multi nominal regression of predictors of PNR and PPR in breast cancer women with and without concomitant diabetes.

Pathological responses	OR (95% CI)	P-value
**PCR**	**Reference**	
**PPR**		
**Age**		
>50	1.42(0.93-2.25)	0.128
≤50	ref	
**BMI**		
Obese	0.91(0.52-1.58)	0.740
Overweight	0.85(0.49-1.50)	0.593
Normal weight	ref	
**Clinical stage**		
IIB	0.97(0.45-2.07)	0.942
IIIA	1.33(0.63-2.81)	0.453
IIIB	**2.71(1.22-5.99)**	**0.014**
IIIC	1.45(0.47-4.44)	0.512
IIA	ref	
**Molecular subtype**		
Luminal B	0.56(0.30-1.03)	0.064
HER2	0.47(0.23-0.93)	0.030
TNBC	0.36(0.21-0.60)	**0.001**
Luminal A	ref	
**Trajectories**		
Consistently hyperglycemia	1.16(0.64-2.09)	0.613
Uncontrolled diabetes	0.92(0.52-1.64)	0.792
Controlled diabetes	1.19(0.59-2.12)	0.731
Merged normal & erratic glycemic instability	ref	
**PNR**		
**Age group** >50	**1.90(1.11-3.24)**	**0.018**
≤50	ref	
**BMI**		
Obese	**2.81(1.36-5.80)**	**0.005**
Overweight	1.55(0.72-3.32)	0.258
Normal weight	ref	
**Clinical stage**		
IIB	0.84(0.36-1.97)	0.693
IIIA	0.80(0.34-1.89)	0.621
IIIB	1.24(0.50-3.05)	0.640
IIIC	**5.83(1.85-18.30)**	**0.003**
IIA	**ref**	
**Molecular subtype**		
Luminal B	0.75(0.36-1.56)	0.452
HER2	0.87(0.39-1.89)	0.727
TNBC	0.501(0.27-0.93)	**0.029**
Luminal A	**ref**	
**Trajectories**		
Group III	1.16(0.55-2.40)	0.690
Group IV	0.72(0.35-1.49)	0.379
Group V	1.75(0.84-3.63)	0.054
Merged Group I and Group II	**ref**	

*Abbreviation: BMI = Body mass index, HER2 = Human epidermal growth factor receptor 2, TNBC = Triple negative breast cancer

### 4.3. Glycemic trajectories in breast cancer women without concomitant diabetes during neo-adjuvant chemotherapy cycles

The dynamic changes in fasting blood glucose levels from cycle 1 to cycle 8 of chemotherapy in the group I,III,II (n = 23, 127,23.2%). In this trajectory group of patients (II,III), fasting blood glucose values were above 126 mg/dL during the chemotherapy.

The fluctuations in fasting blood glucose (FBG) levels in the group I, III,II as shown in Fig 1. Glycemic trajectories in breast cancer women without diabetes during neo-adjuvant chemotherapy cycles.

### 4.4. Glycemic trajectories in breast cancer women with diabetes during neo-adjuvant chemotherapy cycles

The dynamic changes in fasting blood glucose (FBG) levels in group V patients (n = 130) from cycle 1 to cycle 8 of chemotherapy. Only four patients’ FBG values were unstable and fluctuated more than others.

**Fig 2:**
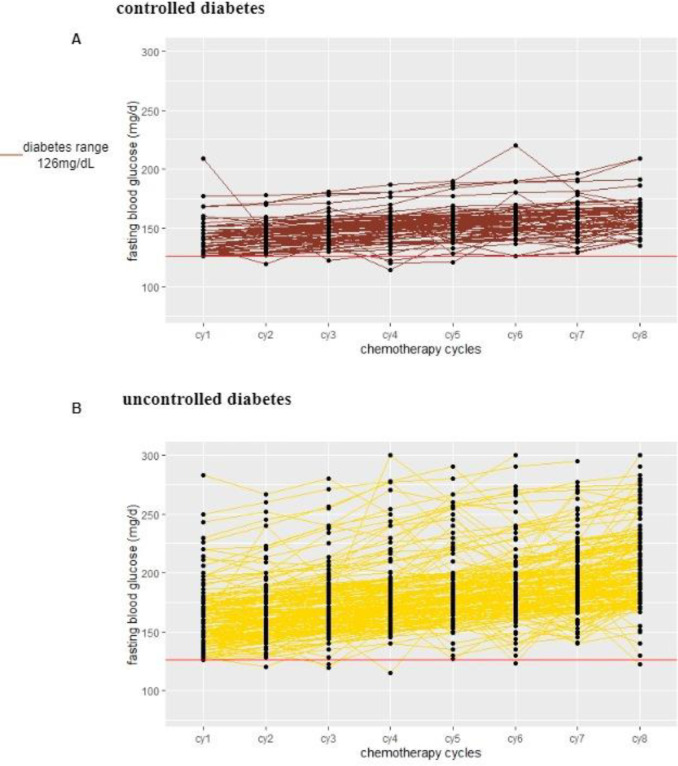
Glycemic trajectories in breast cancer women with diabetes at baseline and end of chemotherapy cycle (A) Controlled diabetes (B) Uncontrolled diabetes.

In group IV breast cancer women (n = 150), most patients’ blood glucose values are above 180 mg/dL. The majority of patients fasting blood glucose values are continuously high after receiving the first cycle of chemotherapy as shown in Fig 2.

### 4.5. Longitudinal Analysis of Chemotherapy Cycles on Fasting Blood Glucose (measured before every Chemotherapy Cycle) and the Interaction with Pathological Responses in Breast Cancer Women with Different Blood Glucose Trajectories Groups

In this study, diabetes and non-diabetes breast cancer patients were categorized into different blood glucose trajectories. Table 4 represent the longitudinal changes in blood glucose trajectories by using statistical analysis one way repeated measure ANOVA.

**Table 4:** Changes in mean fasting blood glucose summary scores: one-way repeated measure ANOVA analysis in diabetes and without diabetes trajectories groups.

Group I	Mean ± SD	p-value
C1	87.87 ± 7.26	<0.05
C2	91.17 ± 6.41	
C3	94.39 ± 6.28	
C4	98.61 ± 6.47	
C5	99.52 ± 4.77	
C6	102.43 ± 7.82	
C7	103.83 ± 8.77	
C8	107.74 ± 8.49	
**Interaction** FBG*pathological responses		>0.05
**Group III**	**Mean ±SD**	**p-value**
C1	109.52 ± 6.281	<0.05
C2	113.12 ± 6.657	
C3	115.65 ± 7.816	
C4	117.66 ± 7.720	
C5	120.70 ± 9.481	
C6	122.72 ± 9.299	
C7	124.50 ± 9.477	
C8	127.42 ± 9.798	
**Interaction** FBG*pathological responses		>0.05
**Group II**	**Mean± SD**	**p-value**
C1	97.96 ± 6.77	<0.05
C2	101.34 ± 6.98	
C3	104.30 ± 5.89	
C4	107.45 ± 5.65	
C5	111.68 ± 5.56	
C6	114.23 ± 7.70	
C7	118.15 ± 8.19	
C8	121.55 ± 8.52	
**Interaction** FBG*pathological responses		<0.05
**Group V**	**Mean±** **SD**	**p-value**
C1	145.72 ± 22.77	<0.05
C2	148.99 ± 22.12	
C3	152.35 ± 23.15	
C4	155.91 ± 22.60	
C5	158.17 ± 18.87	
C6	160.02 ± 15.54	
C7	161.52 ± 16.24	
C8	164.81 ± 15.61	
**Interaction** FBG*pathological responses		>0.05
**Group IV**	**Mean± SD**	**p-value**
C1	161.77 ± 18.66	<0.05
C2	167.37 ± 20.84	
C3	173.86 ± 20.83	
C4	182.25 ± 25.27	
C5	189.27 ± 27.27	
C6	193.73 ± 27.02	
C7	199.95 ± 27.53	
C8	209.89 ± 30.07	
FBG*pathological responses		>0.05

C1 and C2 represent the chemotherapy cycles

#### 4.5.1. Group I. (Normal glycemic trajectory).

See Table 4. In normal glycemic trajectory the fluctuation in Fasting blood glucose was not more as compared to other trajectories.The interaction between FBG and pathological responses show insignificant association.

#### 4.5.2. Group III. (Consistently hyperglycemia).

In consistently hyperglycemia, fasting blood glucose values change in every chemotherapy cycle as shown in Table 4. The interaction between FBG and pathological responses shows an insignificant association(p > 0.05).

#### 4.5.3. Group II. (Erratic glycemic instability).

In the erratic glycemic instability group, fasting blood glucose values change in every chemotherapy cycle. The changes in FBG in every chemotherapy cycle depend on the pathological responses as described in Table 4.

#### 4.5.4. Group V. (Uncontrolled diabetes).

In the group V, the means values of fasting blood glucose change in every chemotherapy cycle. The changes in FBG do not depend on the pathological responses.

#### 4.5.5. Group IV. (Controlled diabetes).

In the group IV, mean fasting blood glucose values change in every chemotherapy cycle. The interaction effect shows that changes in FBG do not depend on pathological responses.

## 5. Discussion

A total of 560 women with breast cancer were included in the study. Among them, 280 (50.0%) had diabetes, while the remaining 280 did not have concomitant diabetes.

Regarding the impact of blood glucose trajectories on pathological response, previous retrospective cohort studies have shown that fasting blood glucose (FBG) variability is independently associated with treatment outcomes in patients with diabetes. However, more prospective trials are needed to confirm the causal relationship between FBG variability and treatment outcomes [9]. To our knowledge, this is the first study in breast cancer patients to report the variability in blood glucose levels and examine its association with pathological responses. These findings may provide valuable insights into the potential role of glycemic variability in predicting treatment responses, particularly in a population undergoing chemotherapy.

In our study, among breast cancer patients with *group V*, some experienced notable spikes in fasting blood glucose, particularly in later chemotherapy cycles (e.g., cycles 6–8). This suggests that, for a subset of patients, chemotherapy may impair glycemic control over time, even in those with previously well-managed diabetes. These findings indicate that patients with *group V* undergoing neo-adjuvant chemotherapy (NACT) may require closer monitoring or more intensive glycemic management as they progress through later chemotherapy cycles. [19].

In patients with *group IV*, the rising trend in blood glucose levels across chemotherapy cycles suggests that chemotherapy may exacerbate glycemic control issues in this population. This group may be more vulnerable to the metabolic effects of chemotherapy, leading to worsened hyperglycemia as treatment progresses. *Group IV* is associated with a higher risk of complications, including infections, delayed wound healing, and more severe chemotherapy side effects.

Compared to the *group V*, patients with *group IV* start with higher fasting blood glucose levels and show more pronounced increases over time. This underscores the difficulty of maintaining glycemic control in patients who already have poorly controlled diabetes before beginning chemotherapy. The variability in fasting blood glucose is also more prominent in the *group IV.* The rising trend and variability in blood glucose suggest that more aggressive interventions, such as insulin therapy or adjustments in diabetes management, may be necessary to avoid complications in this group [20].

Patients in the *group I* maintain normal glucose metabolism during chemotherapy without significant disruptions in fasting blood glucose levels. This indicates that chemotherapy is not causing significant glycemic disturbances in these individuals. In contrast, for patients with *group III*, there may be a predisposition to high blood glucose levels, even in the absence of a formal diabetes diagnosis. The persistence of hyperglycemia during chemotherapy suggests the need for regular monitoring to prevent further increases that could heighten the risk of complications [21]. This group represents patients with *group II* control during chemotherapy, likely due to multifactorial causes such as stress responses, the effects of medications, or other metabolic disruptions induced by chemotherapy. These patients are at higher risk for both hyperglycemia and hypoglycemia, necessitating close glucose monitoring to manage fluctuations effectively [22].

Even among patients without a diabetes diagnosis, glycemic patterns vary. Some maintain normal glycemic control, while others experience persistent hyperglycemia or erratic fluctuations. Patients in the *“group III” and “group II”* are at increased risk for metabolic disturbances and would benefit from close glucose monitoring during chemotherapy. Dietary adjustments, lifestyle counseling, or pharmacological interventions may be necessary to maintain glycemic control in these patients [22].

In contrast, those in the *“group I”* may not require intensive monitoring but should still be observed for any changes, especially as chemotherapy progresses. Identifying these distinct glycemic trajectories can help tailor the level of monitoring and intervention needed. For instance, patients with *group II and group III* fasting blood glucose (FBG) levels may require more aggressive interventions to prevent complications and improve treatment outcomes during chemotherapy.

The timing of significant glycemic variability among breast cancer patients undergoing chemotherapy without concomitant diabetes can be observed starting from Cycle 3, particularly in patients with *group II.* While fasting blood glucose (FBG) levels remain relatively stable in the early chemotherapy cycles (Cycles 1–2), This trend continues and escalates during the mid-cycles (Cycles 3–5), suggesting that from Cycle 3 onward, increased glucose monitoring and intervention may be necessary to manage emerging glycemic variability. However, the most significant variability is observed during the late cycles, where fluctuations become more pronounced, particularly in the *group II*. Some patients in this group experience FBG levels exceeding 200 mg/dL, indicating a considerable risk for hyperglycemia-related complications. [23].

A study done by Mahin et al. reported that steroid-inducing hyperglycemia (SIH) was transient in over 90% of patients, with only seven patients remaining hyperglycemic after completing both glucocorticoid and chemotherapy treatments [24]. This suggests that while glucocorticoid-induced spikes in blood glucose levels are common, for most patients, these effects subside once the steroid treatment is stopped. In comparison, our data, particularly from patients in the “*group II*” and *“group III”*, suggest that some individuals experience prolonged and significant glycemic fluctuations, particularly during the later chemotherapy cycles. The inconsistency in these findings may indicate that while SIH resolves for most patients, a subgroup—such as those with pre-existing metabolic instability or poor baseline glycemic control—remains at risk for persistent hyperglycemia. This aligns with the other study’s finding that a small percentage of patients remain hyperglycemic after therapy [21].

Several factors contribute to the increased blood glucose during the later chemotherapy cycles. One key factor is the cumulative effects of chemotherapy on glucose metabolism [25]. Prolonged exposure to chemotherapeutic agents can disrupt normal metabolic processes, potentially leading to impaired insulin sensitivity or altered glucose homeostasis. In our study, the later cycles show pronounced glycemic variability, which could be partially attributed to the cumulative effects of dexamethasone premedication combined with ongoing chemotherapy-induced metabolic stress. Corticosteroids are known to raise blood glucose levels, and their cumulative use over multiple cycles may intensify hyperglycemia [26]. Furthermore, nutritional status and appetite changes during chemotherapy can also increase variability. As chemotherapy progresses, patients often experience nausea, vomiting, or a reduced appetite, resulting in inconsistent carbohydrate intake, which can destabilize blood glucose control. Additionally, decreased physical activity during later cycles, as patients experience greater fatigue and weakness from the cumulative effects of treatment [27], can exacerbate insulin resistance and further contribute to glycemic variability. These factors collectively highlight the complex interplay between chemotherapy and metabolic control, necessitating careful monitoring and individualized management as patients’ progress through their treatment cycles. Clinical management should be on timely identification and close monitoring during active treatment, particularly for those showing early glycemic lability [23].

Our study demonstrated that non-diabetic patients had significantly lower baseline fasting blood glucose (FBG) levels (102.61 mg/dL) compared to diabetic patients (128.38 mg/dL), which is expected given the impaired glucose regulation in diabetes. Both groups exhibited a significant rise in FBG throughout chemotherapy. The non-diabetic group, starting from a lower baseline, reached 122.96 mg/dL by Cycle 8, while the diabetic group showed a more pronounced increase, reaching 155.75 mg/dL by the same cycle. This steeper rise in FBG among diabetic patients suggests that pre-existing diabetes exacerbates the effects of chemotherapy on glycemic control.

In both the diabetic and non-diabetic groups, FBG increases became statistically significant from Cycle 4 onward, indicating that chemotherapy, particularly after Cycle 3, may contribute to rising blood glucose levels regardless of diabetes status. In the non-diabetic group, we observed an interaction between FBG changes and pathological responses, suggesting that tumor-related factors or treatment responses may influence glycemic control in these patients [28].

In contrast, the diabetic group did not exhibit significant interaction between FBG and pathological responses. This implies that the rise in blood glucose levels in diabetic patients is more likely driven by their pre-existing metabolic dysfunction rather than their pathological response to chemotherapy [28].

In this study most of the breast cancer patients were overweight or obese, and obesity was found to correlate with pathological responses. Many of the obese patients were treated with neo-adjuvant chemotherapy (NACT) along with glucocorticoids, which contributed to dynamic changes in blood glucose levels during chemotherapy. These findings are consistent with previous literature, which has also reported similar metabolic disruptions during cancer treatment [29]. Additionally, our results align with previous retrospective analyses that indicated worse outcomes for breast cancer patients with both high blood glucose levels and high body mass index (BMI) over extended follow-up periods [30]. In terms of pathological responses, the present study shows a variation in breast cancer outcomes based on age distribution. Patients over 50 years of age demonstrated a lower rate of pathologic complete response (pCR) compared to partial pathologic response (pPR) and no pathologic response (pNR). This finding is consistent with the study by Hsu-Huan Chou et al., who reported that younger age is a predictor of pCR, supporting our study results [31]. In our population, most patients achieved pPR, which aligns with previous research [15].

Additionally, we found that more obese patients achieved pPR and pNR rather than complete response. This observation is consistent with previous studies, which have suggested that obesity is not a significant predictor of PCR [32]. Given these findings, it is suggested that personalized strategies such as neo-adjuvant chemotherapy (NACT) dosing may need to be adjusted based on obesity to improve treatment outcomes in this population.

A key strength of this study lies in its contribution of novel insights to the field of oncology, advancing current research perspectives and understanding. The research addresses critical issues related to the impact of blood glucose trajectories on pathological responses in women with breast cancer who have received neo-adjuvant chemotherapy. This research commendably makes a significant contribution, particularly in the context of current healthcare practices at the Gujranwala Institute of Nuclear Medicine and Radiotherapy (GINUM) in Gujranwala, Pakistan.

This study has some limitations. First, the sample size was inadequate for subgroup analysis, so it could not completely explain the stability of results across various trajectory groups. Second, the median follow-up time was short; the conclusion may be more robust with an increased follow-up time of at least 2 years.

## 6. Conclusion

Our study has demonstrated that fluctuations in FBG levels in individuals with diabetes were not significantly associated with pathological responses to neo-adjuvant chemotherapy. Although long-term prospective trials with large sample size are needed to established the causal relationship between dynamic changes in blood glucose and their impact on pathological responses. Future research should incorporate larger, multi center cohort and advanced longitudinal analytical approaches, such as grouped based trajectory modeling (GBTM) or mixed effects modeling, to more accurately characterize glycemic patterns over time and their clinical implication.

Patients with diabetes suffering from breast cancer should be advised to measure their blood glucose values periodically and maintain an HbA1c below 7% in accordance with established guidelines. Although glycemic fluctuations in blood glucose, were not directly linked to pathological responses in this study, both hyperglycemia and hypoglycemia can adversely affects the physical and psychological well-being of individuals living with diabetes may influence treatment tolerance and overall quality of life. Therefore, integrated oncological and glycemic management strategies should be prioritized while further evidence is generated.

## Supporting information

S1 File Prospective phase data-rearrange. (XLSX)
